# Gross Hematuria following SARS-CoV-2 Vaccination

**DOI:** 10.1155/2023/1225510

**Published:** 2023-03-01

**Authors:** Maryam Sattari, Jourdan Mckinnis, Amir Kazory

**Affiliations:** ^1^Division of General Internal Medicine, University of Florida College of Medicine, Gainesville, FL, USA; ^2^University of Florida College of Medicine, Gainesville, FL, USA; ^3^Division of Nephrology, University of Florida College of Medicine, Gainesville, FL, USA

## Abstract

In the setting of the rapid development of currently available vaccines for coronavirus disease of 2019 (COVID-19), little is known about their less frequent potential side effects. Raising the awareness of clinicians and front-line healthcare workers about the less well-known potential side effects of vaccination is important. We describe the self-limited occurrence of gross hematuria in two elderly men on a combination of aspirin and another antiplatelet or anticoagulant following their second dose of the Moderna COVID-19 vaccination. While the bleeding seems to be self-limited, the long-term course currently remains elusive.

## 1. Introduction

While knowledge regarding epidemiologic features, diagnostic tools, and management strategies for coronavirus disease of 2019 (COVID-19) has been rapidly growing, healthcare systems are still challenged by uncertainties surrounding currently available preventive measures [1]. In December 2020, two messenger RNA (mRNA) COVID-19 vaccines were approved by the Food and Drug Administration (FDA) through the Emergency Use Authorization pathway in the United States [1]. Published safety and efficacy trials reported high effectiveness at preventing COVID-19-related illness after two interval doses, along with limited side effects and a low rate of adverse reactions [1]. The mRNA-1273 vaccine (Moderna Inc., Cambridge, Massachusetts, USA) is a lipid nanoparticle-encapsulated mRNA-based vaccine that encodes the prefusion stabilized full-length spike glycoprotein of the severe acute respiratory syndrome coronavirus 2 (SARS-CoV-2) [1]. While pain at the site of injection, fatigue, and headache are among the most recognized reported adverse events, little is known about the vaccine's less frequent potential side effects in the setting of rapid deployment [2]. As COVID-19 vaccinations are now administered globally on a massive scale, rare temporal adverse events are being reported. We describe the self-limited occurrence of hematuria in two patients one day after receiving a second dose of the Moderna COVID-19 vaccination.

## 2. Case Presentation

### 2.1. Patient A

A 71-year-old man was in his usual state of health when he received his second dose of Moderna COVID-19 vaccination. The next day, he noted frank blood in his urine. He also reported two episodes of epistaxis. The patient's past medical history was significant for a non-ST elevation myocardial infarction about two months prior to presentation, after which he had been on dual antiplatelet therapy (DAPT) with aspirin 81 mg daily and clopidogrel 75 mg daily. He denied current or past tobacco use.

Vital signs were normal, and the physical exam only revealed dried blood in the right nostril. The urine dipstick showed small amounts of blood. Lab results were significant for a high monocyte count of 1,086 cells/*μ*L (reference range 200–950) (Table 1). Further urine studies or immunologic assessments were not performed, but the patient was referred to urology. However, he did not establish relationships with them.

While both hematuria and epistaxis resolved without intervention, the patient's cardiologist recommended continuing aspirin and holding Plavix for five days. After five days, the patient resumed DAPT and has not had any further bleeding episodes to date. His most recent renal function, about eleven months after the presentation, was normal with a serum creatinine of 0.76 mg/dL and an estimated glomerular filtration rate (eGFR) of 91 mL/min/1.73 m^2^.

### 2.2. Patient B

A 72-year-old man was in his usual state of health when he received his second dose of Moderna COVID-19 vaccination. The next morning, he noted “wine-colored” urine. His urine gradually became lighter and was “light pink” by the end of the day. However, he continued to have intermittent dark red urine for the next six days. The only other symptom he endorsed was dull right flank pain.

The patient recalled passing a kidney stone about 22 years prior to the presentation but reported more severe pain at that time. His past medical history was also significant for extensive atherosclerotic cardiovascular disease and atrial flutter. His medications included aspirin (81 mg daily) and rivaroxaban (20 mg daily). While the patient had quit tobacco use 32 years prior to the presentation, he did endorse a 40-pack-year smoking history.

Vital signs were normal, and the physical exam revealed tenderness to deep palpation in the suprapubic region. The urine dipstick showed large amounts of blood. Lab results were significant for mildly elevated levels of INR at 1.2 (reference range 0.8–1.1) (Table 1).

A urological work-up for painless gross hematuria in the setting of a history of tobacco use revealed a nonobstructing 9 mm stone in the proximal left ureter and a nonobstructing punctate right renal stone. Hematuria resolved after cystoscopy with bladder irrigation.

## 3. Discussion

The most frequent adverse reactions of COVID-19 Moderna vaccine reported in clinical trials involving more than 30,000 participants were pain at the injection site, fatigue, headache, myalgia, arthralgia, chills, and nausea/vomiting [2]. Adverse reactions were reported less frequently in older adults over 65 years of age than in younger individuals [2]. COVID-19 Moderna postvaccination bleeding events, including epistaxis, hemoptysis, and vaginal bleeding, have been recorded in the United Kingdom Medicines and Healthcare Products Regulatory Agency (MHRA)'s adverse event reports [2]. Since pharmacovigilance data suggest that thrombocytopenia is a frequent postvaccination observation, one proposed mechanism for these bleeding events has been vaccine-induced thrombocytopenia [3]. Actual COVID-19 infection has also been associated with hematuria. Proposed mechanisms for SARS-CoV-2-mediated hematuria include viral tropism for kidney tissue resulting in tubular obstruction and acute kidney injury by indirect factors (e.g., cytokine-mediated, heme-pigment production) and by a direct viral infection and replication in kidney epithelial cells [4–6].

In patients with preexisting, biopsy-proven IgA nephropathy, the development of gross hematuria shortly following Moderna vaccination with spontaneous resolution has been described [7, 8]. In addition to the relapse of preexisting glomerular disease, a wide range of de novo glomerular diseases has been reported shortly after the administration of COVID-19 mRNA vaccines [8–10]. Gross hematuria has been the most common presentation of reported cases of vaccine-associated IgA nephropathy in the literature, followed by acute kidney injury [8, 10]. In the majority of patients, hematuria was self-limited and seldom required immunosuppression [8, 10]. While the underlying mechanism remains to be elucidated, it has been proposed that mRNA-based vaccines result in more robust and/or nonspecific immune activation that may trigger, unmask, or exacerbate autoimmune processes [8]. According to data from the Centers for Disease Control and Prevention, three cases of nephrolithiasis were reported in association with mRNA-based vaccines [11]. Interestingly, a recent retrospective study in Switzerland did not find a statistically significant association between mRNA-based COVID-19 vaccination and an increased incidence of four common glomerulonephritis types (i.e., IgA nephropathy, pauci-immune necrotizing glomerulonephritis, minimal change disease, and membranous nephropathy) [12]. The authors of this study concluded that most cases of new-onset glomerulonephritis manifesting shortly after COVID-19 vaccination are likely the result of temporal coincidence as opposed to causality. They also commented that their findings could not exclude an effect of vaccination on the development of minimal change disease, but the absolute risk would be very small [12].

Unfortunately, neither one of our patients underwent further urine studies (microscopic urine analyses, quantification of proteinuria), nor a renal biopsy, and therefore, it is not known whether there was any underlying glomerular disease. Furthermore, the association between vaccination and hematuria is only temporal. Similar to the majority of macrohaematuria episodes reported in the literature in association with the COVID-19 vaccination [10], our patients' symptoms began within 2 days after the second vaccination dose. Both patients had normal platelet counts. The hematuria in the patient with nephrolithiasis could certainly have been caused by the kidney stones and his medications. He was on aspirin and rivaroxaban, while the other patient was on aspirin and clopidogrel. Certain medications, such as anticoagulants and antiplatelet agents, may increase the risk of postvaccination bleeding; nevertheless, the bleeding resolved itself in both cases. Furthermore, while recurrent hematuria has been reported in some individuals after subsequent doses of the same vaccine [10], both our patients have since received and tolerated the Moderna vaccine booster.

## 4. Conclusions

We hope that this correspondence will raise the awareness of clinicians and front-line healthcare workers about the possibility of a potential association between new-onset hematuria and mRNA-based COVID vaccines. It is possible that patients and clinicians do not recognize these events as vaccine-related. We strongly encourage reporting these postvaccination events to clarify their true incidence. Moreover, if an association with the vaccine is established and bleeding events remain self-limited, important implications include circumventing the cessation of potentially life-saving medications in patients without persistent hematuria, worsening renal function, or other systemic signs and symptoms. A close follow-up and additional workup are warranted to rule out underlying urologic, nephrologic, or hematologic etiology(ies) with relevant long-term health implications that may have been unmasked by the vaccination. Furthermore, some authors have suggested consideration of an alternative vaccine class for subsequent immunization of patients with vaccine-associated hematuria [10].

## Figures and Tables

**Table 1:** Laboratory results.

Test	Patient A results	Reference range^*∗*^	Patient B results	Reference range^*∗*^
WBC	5.1	3.8–10.8 thousand/*μ*L	10.0	4.0–10.0 thousand/*μ*L
Hemoglobin	15.6	13.2–17.1 g/dL	14.3	13.0–16.5 g/dL
Platelet	218	140–400 thousand/*μ*L	194	150–450 thousand/*μ*L
Absolute monocyte	1,086	200–950 cells/*μ*L	740	200–700 cells/*μ*L
Absolute basophil	51	0–200 cells/*μ*L	110	20–90 cells/*μ*L
BUN	22	7–25 mg/dL	16	6–21 mg/dL
Creatinine	1.12	0.7–1.1 mg/dL	1.03	0.51–1.18 mg/dL
PT	11.5	9.0–11.5 seconds	14.2	9.1–13.5 seconds
PTT	24	23–32 seconds	37	25–38 seconds
INR	1.1	1.1	1.2	0.8–1.1
POC urine blood	Small	Negative	Large	Negative
POC urine protein	Trace	Negative	Negative	Negative

BUN = blood urea nitrogen, INR = international normalized ratio, POC = point of care, PT = prothrombin time, PTT = partial thromboplastin time, WBC = white blood cell count. ^*∗*^The different values of reference ranges for patients A and B reflect the ranges of normal values reported by the separate laboratories.

## Data Availability

The deidentified data that support the findings of this study are available from the corresponding author, (MS), upon reasonable request.
